# Association between stress hyperglycemia ratio and in-hospital mortality in acute myocardial infarction: a dose-response meta-analysis

**DOI:** 10.1186/s12872-026-05845-2

**Published:** 2026-05-06

**Authors:** Wei Wang, Qing Li, Lingling Shu, Xiaoli Wu, Tiantian Cui, Zhuolin Xu, Zhengjin Chen

**Affiliations:** Department of Cardiology, The Third Affiliated Hospital of Sun Yat-sen University, Tianhe road 600, Tianhe district, GuangZhou, Guangdong Province 510630 China

**Keywords:** Stress hyperglycemia ratio, Acute myocardial infarction, Dose–response meta-analysis, Mortality

## Abstract

**Background:**

Stress hyperglycemia ratio (SHR) represents the degree of acute glycemic stress relative to chronic glycemic control. Although SHR has been proposed as a better prognostic marker than absolute glucose levels, its quantitative relationship with in-hospital mortality in acute myocardial infarction (AMI) remains uncertain.

**Methods:**

A systematic search of PubMed, Embase, Web of Science, and the Cochrane Library was conducted up to August 10, 2025. Observational studies reporting the association between SHR and in-hospital mortality in AMI were included. Quality assessment was performed using the Newcastle–Ottawa Scale. Pooled odds ratios (ORs) and 95% confidence intervals (CIs) were calculated with a random-effects model. The dose–response relationships were analyzed using the Greenland and Longnecker method and restricted cubic spline models.

**Results:**

Eleven studies involving 27,343 patients were included. Higher SHR was significantly associated with increased in-hospital mortality (pooled OR = 2.14; 95% CI: 1.74–2.55; I² = 63%; *P* < 0.001). The association remained basically consistent across subgroups and sensitivity analysis. Furthermore, the restricted cubic spline model illustrated a non-linear dose-response association between SHR and in-hospital mortality.

**Conclusions:**

In patients with AMI, an elevated SHR is consistently and non-linearly associated with a higher risk of in-hospital mortality. These findings suggest that SHR may serve as a valuable prognostic tool for early risk stratification, although further prospective studies are needed to confirm its clinical utility.

**Supplementary Information:**

The online version contains supplementary material available at 10.1186/s12872-026-05845-2.

## Introduction

Acute myocardial infarction (AMI) remains one of the leading causes of morbidity and mortality worldwide despite substantial advances in reperfusion therapy, pharmacologic management, and intensive cardiac care [1, 2]. A considerable proportion of patients with AMI experience stress-induced hyperglycemia, even in the absence of previously diagnosed diabetes [3]. This acute metabolic response reflects a complex interplay of neuro-endocrine activation, catecholamine surge, elevated cortisol levels, and systemic inflammatory cytokine release [4, 5]. Such transient hyperglycemia may aggravate myocardial injury by increasing oxidative stress, impairing endothelial function, and promoting platelet activation and microvascular obstruction [6, 7]. Consequently, stress hyperglycemia has been proposed as both a marker and a mediator of adverse in-hospital outcomes following AMI.

Traditionally, admission blood glucose and fasting plasma glucose levels have been used to assess the impact of dysglycemia in cardiovascular emergencies. However, these absolute glucose measurements fail to differentiate between chronic hyperglycemia—typically present in diabetic individuals—and the acute glycemic rise attributable to physiological stress [8]. Similarly, glycated hemoglobin (HbA1c) alone reflects long-term glycemic control but does not capture short-term fluctuations during acute illness [9]. These limitations contribute to the inconsistent and sometimes contradictory results observed across studies evaluating glucose levels and AMI prognosis.

To address these shortcomings, the Stress Hyperglycemia Ratio (SHR) was introduced as a dynamic index that integrates both acute and chronic glycemic components [10]. The SHR is calculated as the ratio of admission glucose to estimated average glucose derived from HbA1c [10, 11]. While admission blood glucose (ABG) is a traditional marker of metabolic stress, it is inherently confounded by a patient’s chronic glycemic background. Distinguishing acute stress-induced surges from chronic hyperglycemia is clinically paramount; for instance, aggressive insulin therapy in patients with high baseline HbA1c based on absolute ABG values can lead to iatrogenic hypoglycemia, which paradoxically worsens myocardial injury. Consequently, SHR has emerged as a physiological ‘correction’ tool to isolate the impact of acute stress-induced metabolic derangement from pre-existing glucose levels. This parameter represents the degree to which an individual’s acute glucose level exceeds their chronic glycemic baseline, thereby quantifying the relative intensity of the stress response [10, 11]. Accumulating evidence has shown that elevated SHR is associated with higher rates of in-hospital mortality, heart failure, and major adverse cardiovascular events among AMI patients, regardless of diabetes status [12–14]. Nonetheless, the magnitude and consistency of this association remain controversial. Differences in SHR definitions, population characteristics, follow-up duration, and statistical adjustments across studies have led to considerable heterogeneity in reported outcomes.

Although several observational investigations have explored the prognostic significance of SHR in AMI, important questions remain unresolved [12, 15, 16]. In particular, whether there exists a dose–response relationship between SHR and in-hospital mortality has not been clearly established. Most prior reports dichotomized or categorized SHR values into tertiles or quartiles, limiting the ability to estimate incremental risk associated with each unit increase in SHR. To date, no meta-analysis has comprehensively synthesized the evidence on this quantitative relationship.

Therefore, we conducted a comprehensive systematic review and dose-response meta-analysis to quantify the association between SHR and in-hospital mortality among patients with AMI. By elucidating the quantitative impact of SHR on early mortality, this study aims to enhance understanding of the prognostic role of SHR in AMI and to inform potential refinements in glycemic management strategies during the critical phase of hospitalization.

## Materials and methods

This systematic review and meta-analysis was conducted in accordance with the Meta-analysis of Observational Studies in Epidemiology (MOOSE) statement and followed the PRISMA 2020 reporting guidelines [17, 18]. Two investigators (WW and LQ) independently performed search strategy, study selection, data extraction, quality assessment, and statistical analysis. Any discrepancies were resolved through discussion or consultation with a third reviewer (CZJ).

### Data sources and search strategy

A comprehensive search of four electronic databases—PubMed, Embase, Web of Science, and the Cochrane Library—was performed from database inception to August 10, 2025. The search strategy combined both Medical Subject Headings (MeSH) and free-text terms related to SHR, AMI, and mortality. The following keywords were used in various combinations with Boolean operators (“AND,” “OR”):“stress hyperglycemia ratio,” “stress-induced hyperglycemia,” “acute myocardial infarction,” “myocardial infarction,” “ST-elevation myocardial infarction,” “non-ST-elevation myocardial infarction,” “mortality,” “death,” and “outcome.” To ensure literature completeness, we manually screened the reference lists of all relevant articles and prior reviews, and cross-checked citations of key studies. We also searched ClinicalTrials.gov to identify ongoing or unpublished studies. No language, publication year, or regional restrictions were applied. All retrieved records were imported into EndNote X20 for duplicate removal and systematic screening.

### Study selection and eligibility criteria

Study selection strictly adhered to predefined eligibility criteria established prior to data extraction. Studies were considered eligible if they met all of the following criteria:

#### Population

Adult patients (≥ 18 years) diagnosed with AMI, including both ST-segment elevation myocardial infarction (STEMI) and non-ST-segment elevation myocardial infarction (NSTEMI).

#### Exposure

SHR was defined in accordance with the criteria adopted by the included studies.

#### Comparator

Lower SHR categories or the lowest quantile/tertile/quartile, serving as the reference group.

#### Outcome

In-hospital all-cause mortality reported as odds ratios (ORs), relative risks (RRs), or hazard ratios (HRs) with corresponding 95% confidence intervals (CIs), or sufficient data to calculate these effect sizes.

#### Study design

Observational studies (prospective or retrospective cohort, or case–control) providing at least two quantitative SHR categories.

Exclusion criteria were as follows:

(i)  studies that enrolled mixed populations (e.g., sepsis, stroke, cardiac surgery, or ICU patients) without separate AMI-specific results;
(ii)  studies that did not report in-hospital mortality or failed to provide effect estimates or raw data for computation;
(iii)  abstracts, editorials, reviews, case reports, or conference proceedings without original data; and.
(iv)   duplicate publications using overlapping cohorts, in which case the most complete or recent dataset was retained.

### Data extraction

For each study, the following information was recorded, including study characteristics(first author, publication year, country or region, study design, data source, sample size, and duration of follow-up), participant characteristics (mean or median age, proportion of males, prevalence of diabetes mellitus, and baseline comorbidities), exposure assessment (definition and calculation formula of SHR, the number and boundaries of SHR categories, and the corresponding SHR values used for dose-response modeling), and outcome measures (number of deaths, adjusted and unadjusted effect estimates (OR, RR, or HR) with 95% CIs for in-hospital all-cause mortality, and covariates included in multivariable models). When both unadjusted and adjusted estimates were available, the most fully adjusted model was extracted to minimize confounding. If effect sizes were reported separately for subgroups (e.g., diabetic vs. non-diabetic), data were extracted independently for each subgroup. For studies reporting risk estimates across multiple SHR categories, the natural logarithm of each OR (logOR) and its corresponding standard error (SE) were calculated. To address the ‘heterogeneity in calculation methods,’ we categorized SHR definitions into two main types: (1) the glucose-to-estimated-average-glucose (eAG) ratio, which utilizes the formula: Admission Glucose / [1.59 × HbA1c – 2.59]; and (2) the direct glucose-to-HbA1c ratio. Furthermore, we recorded whether studies used admission random glucose or fasting plasma glucose as the numerator, ensuring that these methodological variations were considered in our assessment of inter-study heterogeneity.

### Quality assessment

The methodological quality of each included study was independently evaluated using the Newcastle–Ottawa Scale (NOS), which assesses the risk of bias across three major domains: selection of participants (4 items), comparability of study groups (2 items), and ascertainment of outcomes (3 items) [19]. A maximum of nine stars could be awarded to each study, with higher scores indicating better methodological quality. Studies were categorized as high quality (7–9 stars), moderate quality (4–6 stars), or low quality (0–3 stars).

### Statistical analysis

Pooled associations between SHR and in-hospital mortality were estimated using random-effects models based on the DerSimonian–Laird method. Between-study heterogeneity was quantified with the Cochran Q statistic and I² statistic, with values of 25%, 50%, and 75% indicating low, moderate, and high heterogeneity, respectively [20]. A *p* value < 0.10 for the Q test was considered statistically significant heterogeneity [21]. Prespecified subgroup analyses were conducted to explore potential sources of heterogeneity, including study design (prospective vs. retrospective), diabetes status (with vs. without diabetes), study region (Asia vs. non-Asia), study quality (high vs. moderate, based on the Newcastle–Ottawa Scale), and sample size (> 1,000 vs. ≤ 1,000 participants). Furthermore, subgroup analyses based on SHR definitions, timing of glucose measurement were also conducted. To assess the robustness of the pooled estimate, a sensitivity analysis was also performed by sequentially omitting one study at a time (“leave-one-out” approach) and examining its influence on the overall pooled OR.

Publication bias was evaluated by visual inspection of funnel plots and formally tested using the Egger regression test [22]. A *P* value < 0.05 was considered suggestive of small-study effects. When publication bias was suspected, the trim-and-fill method was applied to estimate the potential influence of unpublished studies on the pooled effect estimate [23]. A two-sided *P* value less than 0.05 was considered statistically significant. All statistical analyses were conducted using Stata statistical software, version 14.0 (StataCorp, College Station, TX, USA).

### Dose–response analysis

We used the methods proposed by Greenland and Longnecker to evaluate the dose–response relationship between SHR and in-hospital all-cause mortality [24]. We computed study-specific ORs and 95% CIs from the natural logarithms of the ORs and CIs across categories of SHR. This method requires that the exposure levels of SHR, distributions of cases and noncases, and ORs with 95% CIs be available for at least three quantitative categories within each study. We excluded studies from trend estimation that reported fewer than three quantitative SHR categories or lacked sufficient information for model fitting. The median or mean level of SHR in each category was assigned to the corresponding OR for each study. If the median or mean level was not provided and only ranges were reported, we estimated the midpoint in each category by calculating the average of the lower and upper boundaries of that category. If the highest category was open-ended, the midpoint of the category was set at 1.5 times the lower boundary of that category. If the lowest category was open-ended, the midpoint of the category was set at 0.5 times the upper boundary of that category. All analyses were performed using Stata statistical software, version 14.0 (StataCorp, College Station, TX, USA).

## Results

### Study selection and characteristics

**Fig. 1:**
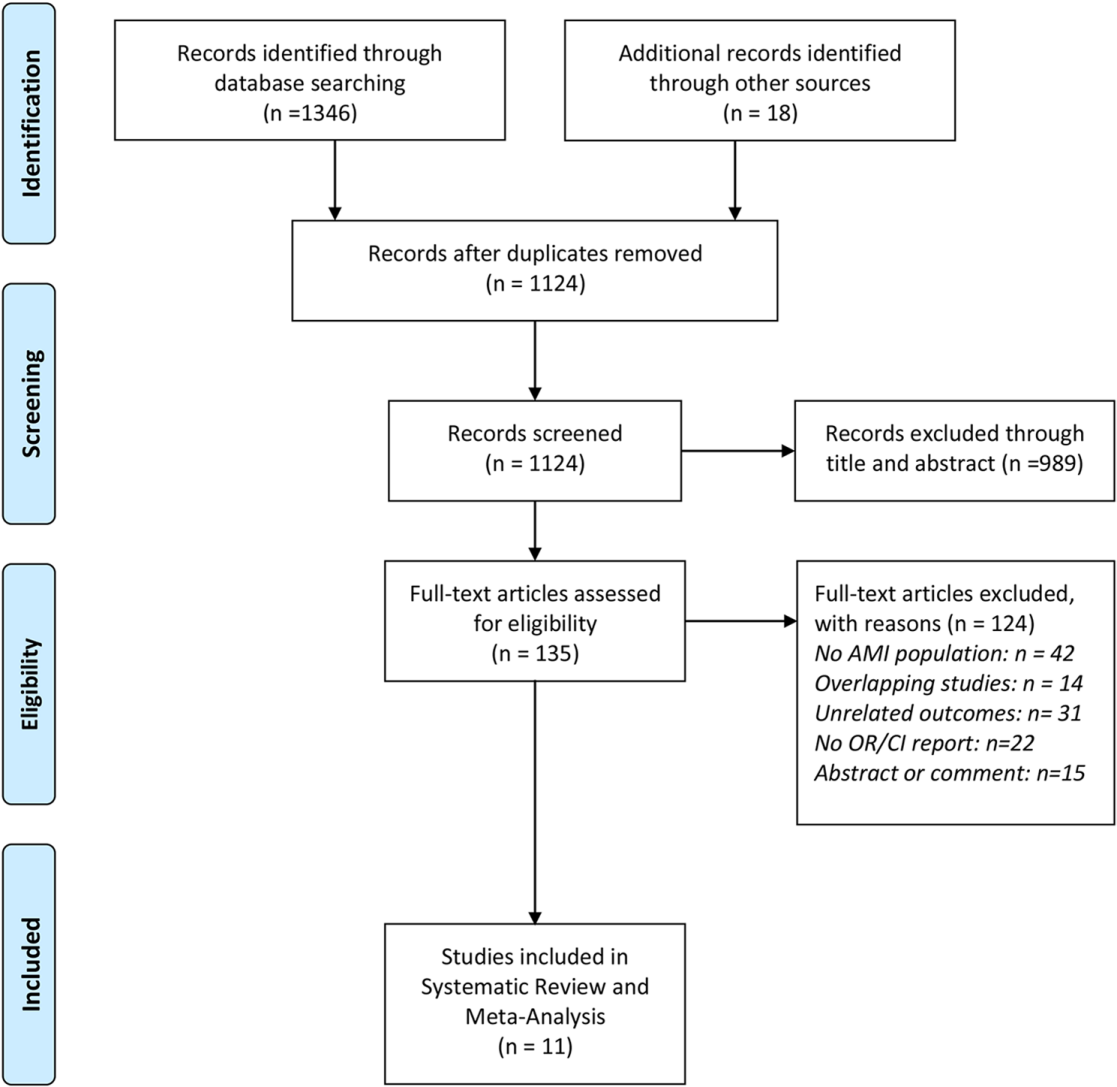
Flow chart of the study selection process for meta-analysis

A total of 1,346 records were identified from database searches, and 18 additional records were obtained through manual searching. After removing duplicates, 1,124 unique records remained for title and abstract screening. Of these, 989 articles were excluded for being irrelevant. After full-text review of 135 potentially eligible studies, 124 were excluded for various reasons, such as non–AMI populations (*n* = 42), duplicate cohorts (*n* = 14), absence of extractable data (*n* = 22), or non-original publications (*n* = 15). Finally, 11 studies met the inclusion criteria and were included in the meta-analysis [12, 15, 16, 25–32] (Fig. 1).

The main characteristics of the included studies are summarized in Table 1. The studies, published between 2018 and 2025, comprised a total of 27,343 patients with AMI. Most studies were retrospective cohorts (*n* = 8), and three were prospective in design (Table 1). The mean or median SHR values varied across studies, with most dividing participants into tertiles or quartiles according to SHR distribution. To better characterize the methodological landscape, we quantified the variability in SHR definitions. Of these included studies, two primary mathematical approaches were identified: ABG/eAG ratio (*n* = 9) [12, 15, 16, 25–28, 31, 32] and FPG/eAG ratio (*n* = 2) [29, 30]. Regarding the temporal aspect of glucose sampling, 9 studies utilized random glucose measured after admission, while 2 study utilized fasting samples collected after admission (Table S1). All studies assessed in-hospital mortality as the primary outcome and the majority of studies adjusted for age, sex, diabetes status, renal function, and key cardiac parameters. The methodological quality of included studies was generally moderate to high, with Newcastle–Ottawa Scale scores ranging from 6 to 9 (Table 2).

**Table 1:** Baseline characteristics of included studies

Author (Year)	Study type	Study center	Country	No. of patients	Population	Mortality	Endpoints	Adjustment for confounders
Marenzi et al. (2018) [15]	Prospective cohort study	Single center	Italy	1,553	Adults with AMI admitted to the Intensive Cardiac Care Unit	1.70%	In-hospital mortality	Age, sex, STEMI, diabetes, hypertension, renal function (eGFR, creatinine), LVEF, smoking, Killip class, prior MI, reperfusion therapy
Gao et al. (2020) [25]	Retrospective cohort study	Single center	China	1,300	Adults with STEMI admitted to the Intensive Cardiac Care Unit	2.30%	In-hospital mortality	Age, gender, peak troponin I, PCI timing (primary vs. delayed), and Gensini score (extent of coronary disease)
Chen et al. (2021) [26]	Retrospective cohort study	Single center	China	341	Aged patients with AMI admitted to CCU	12.90%	In-hospital mortality	Age, sex, diabetes, cardiac arrest before admission, Killip class, heart rate, systolic blood pressure, symptom-to-balloon time (S2B), PCI, complete revascularization, LVEF, NT-proBNP, and admission glucose
Gao et al. (2021) [27]	Retrospective cohort study	Single center	China	1,215	Hospitalized diabetic patients with AMI	4.30%	In-hospital mortality	Age, sex, MI classification, PCI treatment (yes/no), and peak troponin I levels
Xu et al. (2022) [16]	Retrospective cohort study	Multi-center	China	5,417	Adults with acute STEMI	14.60%	30-day mortality	Age, systolic blood pressure, heart rate, Killip class, diabetes, hypertension, angina, weight, anterior STE or LBBB, and time-to-treatment > 4 h
Cui et al. (2023) [29]	Prospective cohort study	Multi-center	China	5,308	AMI patients	3.90%	In-hospital mortality	Age, sex, BMI, STEMI, PCI, smoking, hypertension, prior MI/PCI/stroke, CKD, HR, SBP, LVEF, TG, LDL-C, statin use
Chen Q (2023) [28]	Retrospective cohort study	Multi-center	China	1,732	AMI patients	6.50%	In-hospital mortality	GRACE score, sex, smoking, previous PCI, hypertension, diabetes, uric acid, hypercholesterolemia, inpatient revascularization, coronary complexity, discharge meds (β-blocker, diuretic, statin, ACEI/ARB)
Wei et al. (2023) [31]	Retrospective cohort study	Single-center	China	1,099	Patients with STEMI	6.30%	In-hospital death	Ischemic time, age, sex, BMI, hypertension, hyperlipidemia, diabetes, smoking, CKD, prior CAD, AF, HF, stroke, cancer, culprit vessel, multi-vessel disease
Fu et al. (2023) [30]	Prospective registry-based cohort	Mult-icenter	China	5,308	Patients with AMI	3.50%	In-hospital death	Age, sex, BMI, STEMI, Killip II–IV, primary PCI, smoking, hypertension, prior MI/PCI/stroke, CKD, heart rate, SBP, LVEF, TG, LDL-C, in-hospital statin use
Lai et al. (2025) [12]	Retrospective cohort	Single center	USA	1,933	Patients with AMI admitted to ICU	20.70%	In-hospital mortality	Age, sex, race, diabetes, stroke, HF, AF, CKD, COPD, BMI, SBP, HR, Hb, WBC, PLT, albumin, lactate, creatinine, PaO₂, TG, LDL, HDL, LVEF, SOFA, SIRS, SAPS II/APS III, ACEI/ARB, β-blocker, statin, aspirin, diuretic, CRRT, ventilation, PCI, CABG, IABP
Li et al. (2025) [32]	Retrospective cohort	Single center	USA	4,663	Patients with AMI admitted to ICU	6.90%	In-hospital mortality	Age, BMI, race, diabetes, AF, AHF, STEMI, hypertension, CKD, CKD5, CABG, PCI, SOFA, neutrophils, CKMB, creatinine, BUN, albumin, ALT, AST, TC, HDL, lactate, EF, UO, ACEI/ARB, insulin

**Table 2:** Methodological Quality Assessment of Included Studies by Newcastle–Ottawa Scales

Study	Selection					Outcome			
	Exposed Cohort	Nonexposed Cohort	Ascertainment of Exposure	Outcome of Interest	Comparability	Assessment of Outcome	Length of Follow-up	Adequacy of Follow-up	Total Score
Marenzi G et al. (2018) [15]	*	*	*	*	**	*	*	*	9
Gao et al. (2020) [25]	*	*	*	*	**	*	*	——	8
Chen et al. (2021) [26]	*	*	*	*	**	*	*	——	8
Gao et al. (2021) [27]	*	*	*	*	*	*	*	——	7
Xu et al. (2022) [16]	——	*	*	*	*	*	*	——	6
Cui et al. (2023) [29]	——	*	*	*	*	*	*	*	7
Chen et al.(2023) [28]	*	*	*	*	**	*	*	——	8
Wei et al.(2023) [31]	*	*	*	*	**	*	*	——	8
Fu et al.(2023) [30]	*	*	*	*	**	*	*	——	8
Lai et al.(2025) [12]	*	*	*	*	*	*	*	——	7
Li et al.(2025) [32]	*	*	*	*	**	*	*	——	8

Single asterisk indicates 1 score, double asterisk indicates 2 scores, and dash indicates 0 scores.

### Association between SHR and In-hospital mortality

**Fig. 2:**
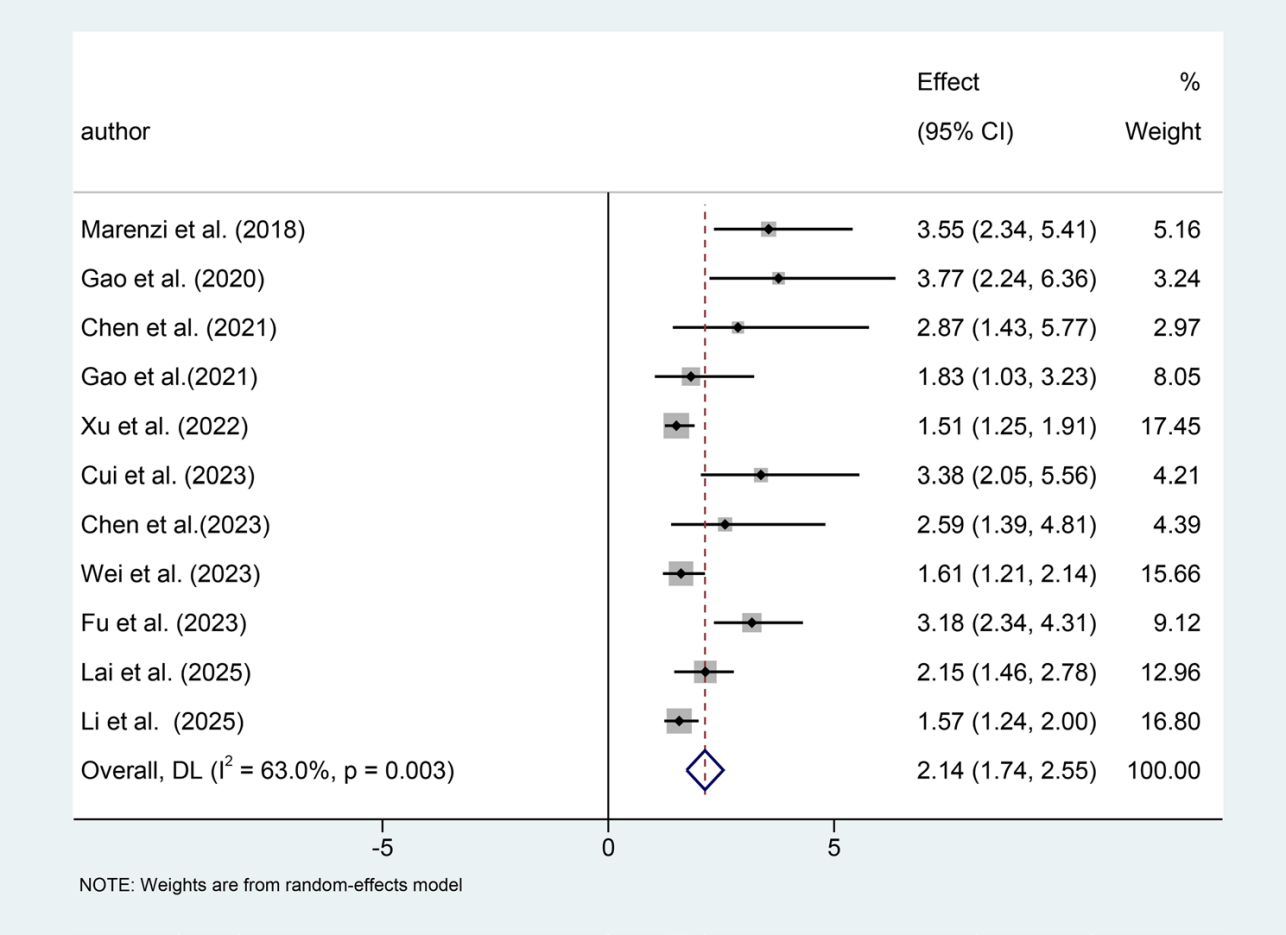
Forest plots for the meta-analysis of the association between stress hyperglycemia ratio and in-hospital mortality in acute myocardial infarction

Pooled estimates showed that a higher SHR was significantly associated with an increased risk of in-hospital mortality among AMI patients (pooled OR = 2.14; 95% CI, 1.74–2.55; I² = 63%) (Fig. 2). The association remained robust in the random-effects model, suggesting that individuals with elevated SHR had approximately a 2-fold higher odds of in-hospital mortality compared with those with lower SHR values. The between-study heterogeneity was substantial, likely reflecting differences in study design, patient characteristics, and the definition of SHR categories.

### Subgroup and sensitivity analyses

We also conducted subgroup (Table 3) and sensitivity analyses to explore the robustness and consistency of the findings. When stratified by study design, the pooled OR was 3.305 (95% CI, 2.555–4.054) for prospective studies and 2.141 (95% CI, 1.735–2.547) for retrospective studies. The association remained significant across regions (Asia vs. non-Asia), sample sizes (≥ 1,000 vs. <1,000 patients), and diabetes subgroups. Both diabetic (OR, 1.974; 95% CI, 1.403–2.777) and non-diabetic patients (OR, 2.219; 95% CI, 1.702–2.894) exhibited a consistent positive association. When categorized by SHR cut-offs, stronger associations were observed in studies using dichotomized groups (OR, 3.230; 95% CI, 2.407–4.052) than in those using tertiles (OR, 2.066; 95% CI, 1.460–2.671) or quartiles (OR, 1.711; 95% CI, 1.229–2.192). Analyses stratified by methodological quality revealed stronger associations in studies with NOS ≥ 8 (OR, 2.319; 95% CI, 1.834–2.804) than those with lower quality scores (OR, 1.507; 95% CI, 1.178–1.836). Analyses stratified by SHR definitions or timing of glucose measurement revealed stronger associations in studies with FPG or FPG/eAG ratio (OR, 3.228; 95% CI, 2.369–4.087) than those with ABG or ABG/eAG ratio (OR, 1.874; 95% CI, 1.529–2.219). Owing to limited data involving infarct severity and statistical approaches, we did not perform subgroup analyses for them. Interestingly, we found that significant subgroup differences (*p* < 0.05) were observed in all four analyses (Study design, SHR grouping, SHR definitions, and NOS score).

**Table 3:** Subgroup analyses of relationship between stress hyperglycemia ratio and in-hospital mortality in acute myocardial infarction

Subgroup	No. Studies	Test of Relationship		Test of Heterogeneity		Between-subgroup heterogeneity
		OR (95% CI)	*P* value	I^2^, %	*P* value	
Total	11	2.141(1.735–2.547)	0	63	0.003	
Study design						
Prospective study	3	3.305(2.555–4.054)	0	0	0.92	**0**
Retrospective study	8	2.141(1.735–2.547)	0	27.5	0.209	
Study center						0.405
Single center	7	2.013(1.555–2.472)	0	52.2	0.051	
Multi center	4	2.538(1.392–3.684)	0	79.2	0.002	
Region						0.846
Asia	8	2.240(1.678–2.801)	0	64.1	0.007	
Europe and America	3	2.141(1.328–2.955)	0	73.3	0.024	
AMI patients						NA
DM	9	1.974(1.403–2.777)	0	81.2	0.001	
Non-DM	9	2.219(1.702–2.894)	0	80.5	0.001	
SHR grouping						**0.008**
Dichotomization	3	3.230(2.407–4.052)	0	0	0.827	
Tertiles	5	2.066(1.460–2.671)	0	58.8	0.045	
Quartiles	3	1.711(1.229–2.192)	0	49	0.141	
SHR definitions or glucose measurement time						**0.004**
FPG or FPG/eAG ratio	2	3.228(2.369–4.087)	0	0	0.846	
ABG or ABG/eAG ratio	9	1.874(1.529–2.219)	0	47.9	0.053	
Sample size						0.41
<1000	2	2.695(1.352–4.038)	0	0	0.84	
>1000	9	2.103(1.676–2.530)	0	68.2	0.001	
NOS score						**0.007**
Moderate	1	1.507(1.178–1.836)	0	NA	NA	
High	10	2.319(1.834–2.804)	0	61.1	0.006	

*ABG* Admission blood glucose, *FPG* Fasting Plasma Glucose, *eAG* Estimated Average Glucose, *SHR* Stress Hyperglycemia Ratio

**Fig. 3:**
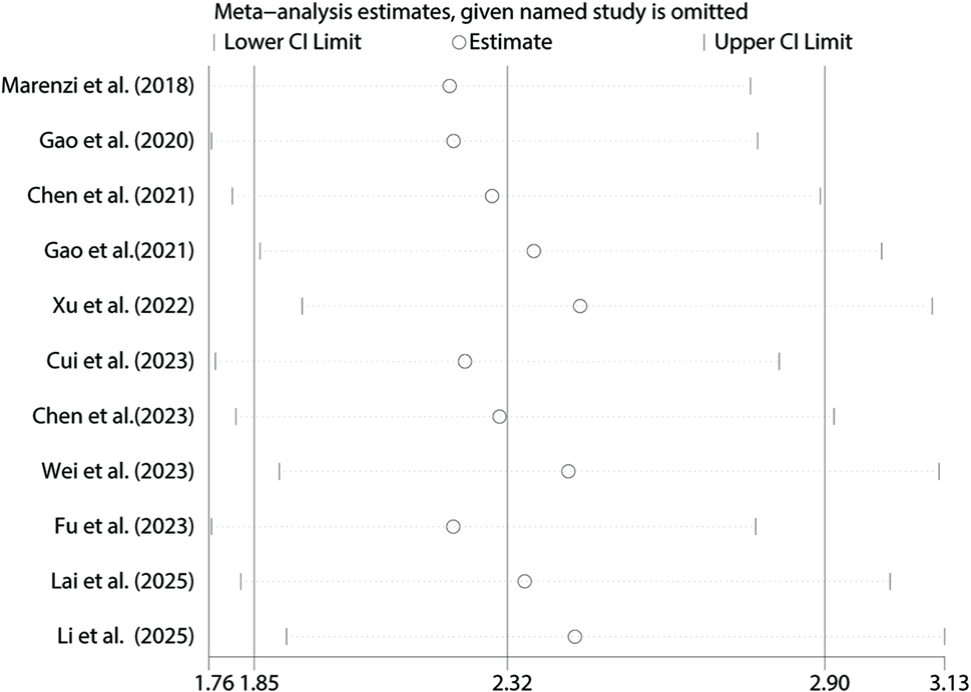
Sensitivity analysis for stress hyperglycemia ratio and in-hospital mortality in acute myocardial infarction

Sensitivity analysis using the leave-one-out method demonstrated the stability of the pooled estimates (Fig. 3). Sequential exclusion of any single study did not materially alter the overall effect size (pooled ORs ranging from 2.20 to 2.45), indicating that no single study disproportionately influenced the overall association.

### Dose–response relationship

**Fig. 4:**
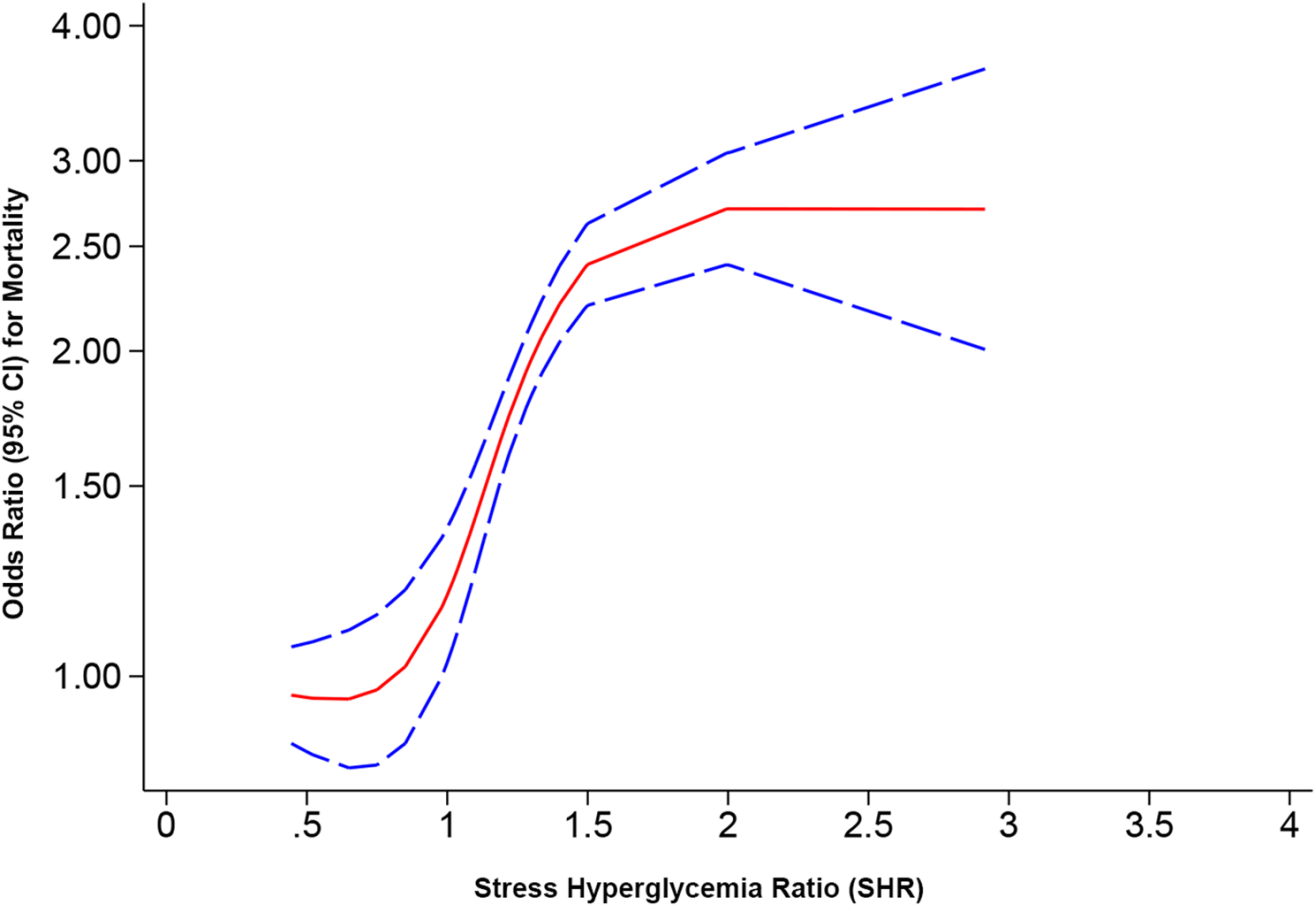
Dose-response plot of stress hyperglycemia ratio and in-hospital mortality in acute myocardial infarction

A total of eight studies were eligible for dose-response meta-analysis and a nonlinear dose–response association between SHR and in-hospital mortality was identified [12, 15, 16, 25, 28, 29, 31, 32]. The restricted cubic spline model illustrated a non-linear dose-response association between SHR and in-hospital mortality. Notably, the risk of mortality remained relatively stable at lower SHR levels but exhibited a steep upward trend as SHR exceeded approximately 1.0 (P for non-linearity < 0.001), suggesting a possible inflection point for clinical risk stratification (Fig. 4). Taken together, these results demonstrate a strong and consistent association between elevated SHR and in-hospital mortality in patients with AMI, with evidence of a nonlinear dose–response relationship that remains robust across multiple sensitivity and subgroup analyses.

### Publication bias

Visual inspection of the funnel plot suggested mild asymmetry (Figure S1). Egger’s regression test detected potential small-study effects (*P* = 0.024). However, after adjustment using the trim-and-fill method, the pooled estimate (OR, 2.00; 95% CI, 1.61–2.49) remained significant and of similar magnitude after adding three potential missing studies’ (Figure S2), indicating that the results were unlikely to be materially affected by publication bias.

## Discussion

### Principal findings

In the dose–response meta-analysis, we observed that elevated SHR was significantly associated with increased in-hospital mortality among patients with AMI. The association remained robust across subgroup and sensitivity analyses, with a clear nonlinear dose–response pattern. These findings provide quantitative evidence that SHR captures a critical metabolic disturbance linked to short-term mortality risk in AMI.

### Comparison with previous evidence

Our results are also consistent with a recent meta-analysis in the neurologic domain, which demonstrated that elevated SHR levels are significantly associated with poor functional recovery following acute ischemic stroke [33]. This suggests that whether the primary insult is myocardial or cerebral, the resulting “metabolic storm” characterized by catecholamine-driven insulin resistance—follows a similar pathological trajectory toward adverse outcomes. Furthermore, the non-linear relationship identified in our dose-response analysis is corroborated by large-scale evidence from critically ill populations [34]. In a meta-analysis encompassing over 102,000 patients, SHR exhibited a consistent positive link with multiple mortality endpoints, mirroring the trend observed in our AMI cohort. By situating our findings within this broader landscape of “relative hyperglycemia,” it becomes evident that SHR is not merely a cardiac-specific marker but rather a systemic indicator of acute physiological exhaustion. Previous studies have documented the adverse impact of stress-induced hyperglycemia on outcomes after cardiovascular events, yet their conclusions have been inconsistent because of heterogeneity in study populations and the use of absolute glucose levels [3, 35, 36]. By integrating data from 11 independent studies and applying formal dose–response modeling, we identified a graded and nonlinear association between SHR and mortality in AMI. Unlike prior meta-analyses that examined admission glucose alone [35, 36], our study adjusted for baseline glycemic status via HbA1c, thereby reducing confounding by chronic diabetes control. Our findings therefore extend earlier observations and delineate a quantifiable threshold beyond which the stress response becomes pathologic.

### Potential mechanisms

Several mechanisms may explain the observed association. On one hand, acute glycemic excursions during AMI are mediated by catecholamine surge, cortisol release, and inflammatory cytokine activation, which collectively augment hepatic gluconeogenesis and inhibit peripheral glucose uptake. Elevated SHR levels may reflect a state of metabolic inflexibility, which is defined as the heart’s diminished capacity to dynamically switch between fatty acid and glucose oxidation to meet energy demands during acute stress. Under normal physiological conditions, the heart maintains high metabolic plasticity; however, during the acute phase of an infarction, profound insulin resistance and catecholamine-driven hyperglycemia impair this transition. This ‘locked’ metabolic state prevents the ischemic myocardium from utilizing glucose efficiently, leading to an accumulation of toxic lipid intermediates and increased oxidative stress, which collectively accelerate cardiomyocyte death. On the other hand, elevated glucose levels promote advanced glycation, increased reactive oxygen species, and microvascular endothelial dysfunction, which impair reperfusion and amplify infarct size. SHR is uniquely positioned to reflect this pathophysiologic stress because it normalizes admission glucose against HbA1c-derived baseline glycemia, identifying patients whose acute glucose elevation is disproportionate to chronic levels. Such “relative hyperglycemia” has been linked to impaired immune competence, enhanced platelet activation, and reduced nitric-oxide bioavailability, all of which contribute to increased mortality independent of diabetes status. The persistence of this association in both diabetic and nondiabetic subgroups underscores the biological plausibility that SHR represents a global index of metabolic stress and neurohormonal activation, not merely poor chronic control.

### Clinical implications

It is essential to clarify that SHR should currently be regarded as a robust prognostic indicator rather than a validated target for therapeutic intervention. When compared to established risk stratification tools like the GRACE score, the SHR offers distinct advantages by capturing the host’s metabolic resilience. While the GRACE score is highly effective at integrating age, heart rate, and creatinine levels, it often fails to account for the acute ‘metabolic storm’ characterized by relative hyperglycemia. By reflecting the neuro-hormonal burden of the acute insult, the SHR acts as a valuable adjunct that provides incremental predictive value beyond traditional hemodynamic and biochemical parameters. Our findings have significant implications for peri-infarction glucose management. Current guidelines often suggest a ‘one-size-fits-all’ absolute glucose target (e.g., < 180 mg/dL), which may be suboptimal for patients with varied chronic glycemic set-points. By adopting an SHR-based approach, clinicians can potentially refine glycemic targets: a high SHR indicates a profound metabolic crisis requiring close monitoring, whereas a low SHR in the presence of high ABG may suggest that the patient is simply at their chronic baseline, where less intensive glucose-lowering may be safer to avoid the detrimental effects of iatrogenic hypoglycemia. This distinction is particularly relevant for non-diabetic patients, in whom even a modest rise in SHR reflects a disproportionately severe stress response compared to their diabetic counterparts.

While our meta-analysis establishes SHR as a robust prognostic marker, the transition from risk stratification to therapeutic intervention requires targeted randomized controlled trials. Future research should specifically compare SHR-stratified glycemic control protocols against conventional absolute-threshold-based care (e.g., maintaining glucose < 180 mg/dL). Additionally, given the association between stress hyperglycemia and coronary microvascular dysfunction, trials focusing on reducing glycemic variability through continuous glucose monitoring (CGM) in the high-SHR subgroup are warranted. Beyond randomized controlled trials, the translation of SHR into routine clinical practice necessitates several key developments. First, standardization of SHR formulas or timing of measurement was essential in further clinical trials. Second, future efforts should focus on recalibrating existing clinical risk scores, such as the GRACE or TIMI scores, by incorporating SHR as a dynamic metabolic variable to improve their discriminative power. Finally, the development of institutional ‘metabolic alert’ protocols—whereby an elevated SHR triggers specific monitoring or specialized endocrinology consultation—could facilitate a more proactive and personalized approach to managing the acute glycemic stress response in myocardial infarction.

### Strengths and limitations

This meta-analysis adheres to rigorous methodological standards, including comprehensive literature retrieval, duplicate data abstraction, and quality assessment using the Newcastle–Ottawa Scale. The application of a dose–response framework allowed quantitative characterization of the exposure-outcome relationship and identification of a biologically meaningful threshold. Nevertheless, several limitations merit acknowledgment. Firstly, the majority of included studies were observational in design, which precludes definitive causal inference. Although most adjusted for major confounders such as age, diabetes, renal function, and infarct severity, residual confounding from unmeasured variables—including infarct size, inflammatory status, reperfusion delay, and in-hospital treatment—cannot be excluded. Additionally, we were unable to perform subgroup analyses based on pre-hospital therapies, exact ischemia time, or chronic glucose-lowering therapy due to insufficient reporting in the original literature. These factors remain potential sources of unmeasured confounding that could influence the precision of the observed dose-response relationship. Secondly, heterogeneity across studies was moderate to high, arising from differences in study design, population characteristics, infarct type (STEMI vs NSTEMI), reperfusion strategy (PCI vs conservative), Killip class, ICU admission and analytic methods. Although we applied a random-effects model and extensive subgroup analyses to mitigate this issue, unexplained heterogeneity remains an inherent limitation of pooled analyses. Also, the observed heterogeneity may be partially attributed to the lack of a universal consensus on the optimal SHR formula and the timing of glucose sampling. While immediate admission glucose reflects the peak catecholamine-driven surge, fasting glucose may better represent sustained metabolic derangement. Despite these differences, the consistency of the association between high SHR and mortality across all included studies suggests that the prognostic power of ‘relative hyperglycemia’ transcends the specific mathematical derivation used. Standardizing the SHR formula remains a priority for future clinical application to ensure cross-study comparability. Thirdly, the dose–response analysis relied on category midpoints and assumed exposure distributions, as most studies reported SHR in ranges rather than continuous values. While we applied validated methods by Greenland and Longnecker and conducted sensitivity analyses for open-ended categories, some degree of exposure misclassification is unavoidable. Moreover, the limited number of studies with ≥ 3 SHR categories may have reduced the precision of the nonlinear curve estimation. Fourth, potential publication and reporting biases cannot be entirely ruled out. Egger’s regression test indicated small-study effects, which may reflect either selective reporting or underlying heterogeneity rather than genuine bias. The trim-and-fill procedure suggested that any missing data were unlikely to alter the direction or significance of our findings, yet these statistical corrections are imperfect substitutes for unpublished null studies. Taken together, these limitations warrant cautious interpretation of our results. However, the consistency of findings across multiple sensitivity and subgroup analyses, the biologic plausibility of the observed relationship, and the reproducibility of the nonlinear trend support the validity and clinical relevance of our conclusions.

## Conclusions

In conclusion, our dose-response meta-analysis indicates a significant non-linear relationship between the SHR and in-hospital mortality in the AMI population. These findings suggest that SHR may serve as a valuable prognostic tool for early risk stratification, although further prospective studies are needed to confirm its clinical utility.

## Supplementary Information

Supplementary Material 1.

Supplementary Material 2: Funnel plots for the meta-analysis of stress hyperglycemia ratio and in-hospital mortality in acute myocardial infarction

Supplementary Material 3: The trim-and-fill method for the meta-analysis of stress hyperglycemia ratio and in-hospital mortality in acute myocardial infarction.

## Data Availability

The data that support the findings of this study are available from the corresponding author upon reasonable request.

## Abbreviations

SHR: Stress hyperglycemia ratio
AMI: Acute myocardial infarction
STEMI: ST-segment elevation myocardial infarction
NSTEMI: Non–ST-segment elevation myocardial infarction
PCI: Percutaneous coronary intervention
HbA1c: Glycated hemoglobin
GRACE: Global Registry of Acute Coronary Events
OR: Odds ratio
RR: Relative risk
HR: Hazard ratio
CI: Confidence interval
NOS: Newcastle–Ottawa Scale
MOOSE: Meta-analysis of Observational Studies in Epidemiology
PRISMA: Preferred Reporting Items for Systematic Reviews and Meta-Analyses
