# Supplementary material for: Association between stress hyperglycemia ratio and in-hospital mortality in acute myocardial infarction: a dose-response meta-analysis
Source: BMC Cardiovasc Disord. 2026 May 6;26:541. doi: 10.1186/s12872-026-05845-2 (PMC13317392; doi:10.1186/s12872-026-05845-2)
Supplement: Supplementary file 1 — Supplementary Material 1. [file 12872_2026_5845_MOESM1_ESM.docx]

| **Table S1: Baseline characteristics of included studies** | | | | | |
| --- | --- | --- | --- | --- | --- |
| **Author (Year)** | **Glucose Measurement Timing** | **SHR Definitions** | **Cutoffs** | **SHR Grouping** | **OR (95% CI) in multivariate regression** |
| Marenzi et al. (2018) | ABG | ABG/eAG ratio | 1.3 | Tertiles | 1.49 (0.92–2.43) for T2 vs T1; 3.55 (2.34–5.41) for T3 vs T1 |
| Gao et al. (2020) | ABG | ABG/eAG ratio | 1.22 | Dichotomized by SHR cutoff 1.13 | • Non-DM: 5.84 (2.50–13.66) • DM: 2.45 (1.24–4.82),  • Overall: 3.77 (2.24–6.36) |
| Chen et al. (2021) | ABG | ABG/eAG ratio | 1.09 | Dichotomized by SHR cutoff 1.25 | 2.871 ( 1.428–5.772) |
| Gao et al. (2021) | ABG | ABG/eAG ratio | 1.08 | Tertiles | 1.83 (1.03–3.23) for T3 vs T1 |
| Xu et al. (2022) | ABG | ABG/eAG ratio | 0.97 | Tertiles | 1.507 (1.253–1.911) for T3 vs T1 |
| Cui et al. (2023) | FPG | FPG/eAG ratio | 1.01 for Non-DM; 1.05 for DM | Quartiles (Q1–Q4) | DM: Q4 vs Q1, OR = 4.070 (2.014–8.228); Continuous: OR = 3.682 (2.380–5.696). Non-DM: Q4 vs Q1, OR = 2.976 (1.695–5.224); Continuous: OR = 1.109 (1.016–1.211) |
| Chen Q (2023) | ABG | ABG/eAG ratio | 1.16 | Tertiles | 2.586 (1.391–4.808) for T3 vs T1 |
| Wei et al. (2023) | ABG | ABG/eAG ratio | 1.51 | Quartiles | SHR1: OR 1.61 (1.21–2.14); SHR2: OR 1.57 (1.22–2.01); SHR3: OR 1.59 (1.24–2.05). |
| Fu et al. (2023) | FPG | FPG/eAG ratio | 1.01 | Dichotomize | DM: 3.159 (1.932–5.165); Non-DM: 3.189 (2.161–4.705) |
| Lai et al. (2025) | ABG | ABG/eAG ratio | 1.14 | Tertiles | 1.16 (0.76–1.76) for T2 vs T1; 2.15 (1.46–2.78) for T3 vs T1 |
| Li et al. (2025) | ABG | ABG/eAG ratio | 1.14 | Quartiles | 1.09 (0.89–1.35) for T1 vs T2; 1.13 (0.91–1.39) for T3 vs T2; 1.57 (1.24–2.00) for T4 vs T2 |
| ABG: Admission blood glucose; FPG; Fasting Plasma Glucose; eAG: Estimated Average Glucose; SHR: Stress Hyperglycemia Ratio | | | | | |
