# Supplementary figures and images for: Association between stress hyperglycemia ratio and in-hospital mortality in acute myocardial infarction: a dose-response meta-analysis
Source: BMC Cardiovasc Disord. 2026 May 6;26:541. doi: 10.1186/s12872-026-05845-2 (PMC13317392; doi:10.1186/s12872-026-05845-2)

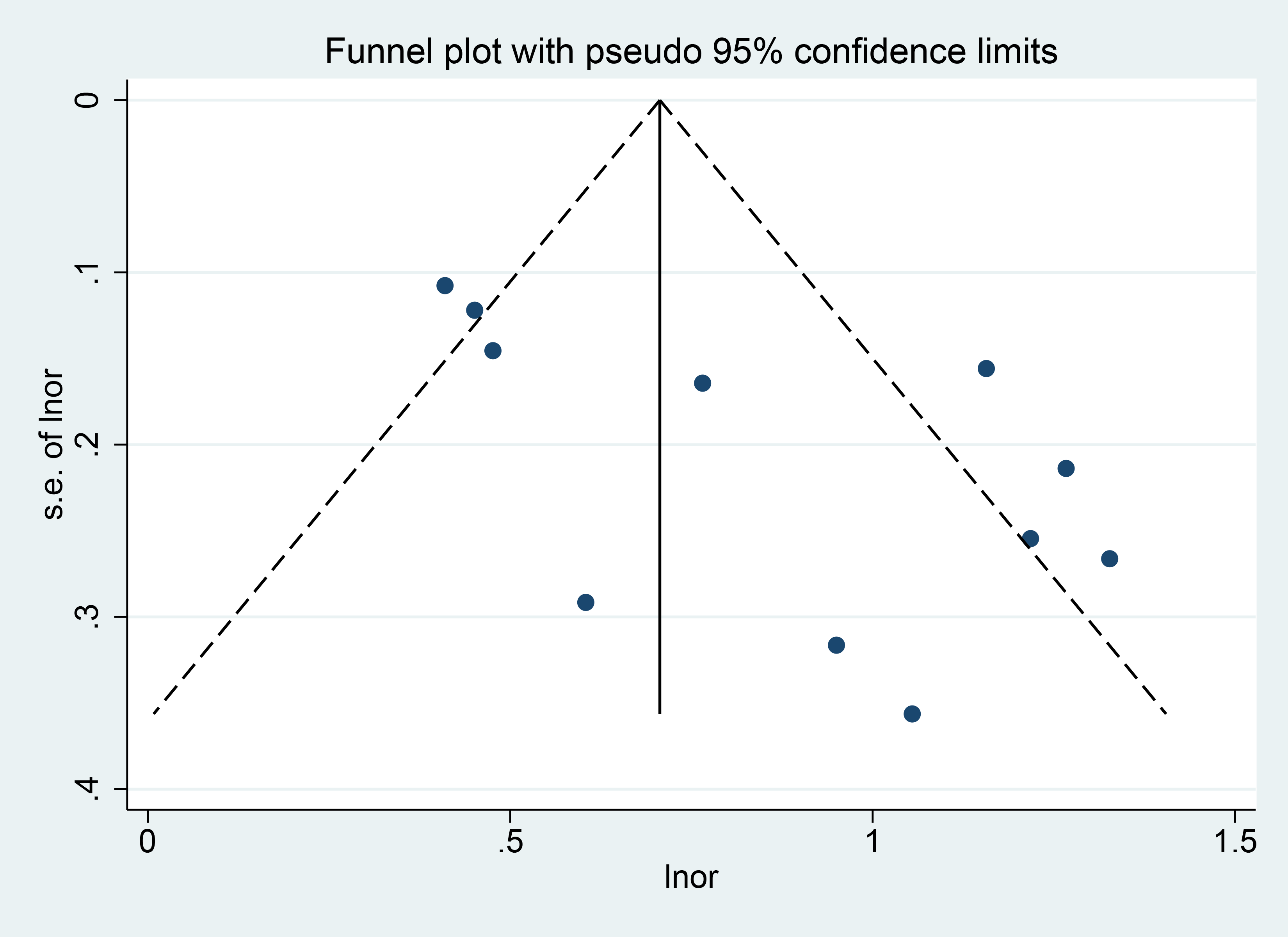

Supplement: Supplementary file 2 — Supplementary Material 2: Funnel plots for the meta-analysis of stress hyperglycemia ratio and in-hospital mortality in acute myocardial infarction [file 12872_2026_5845_MOESM2_ESM.tif]

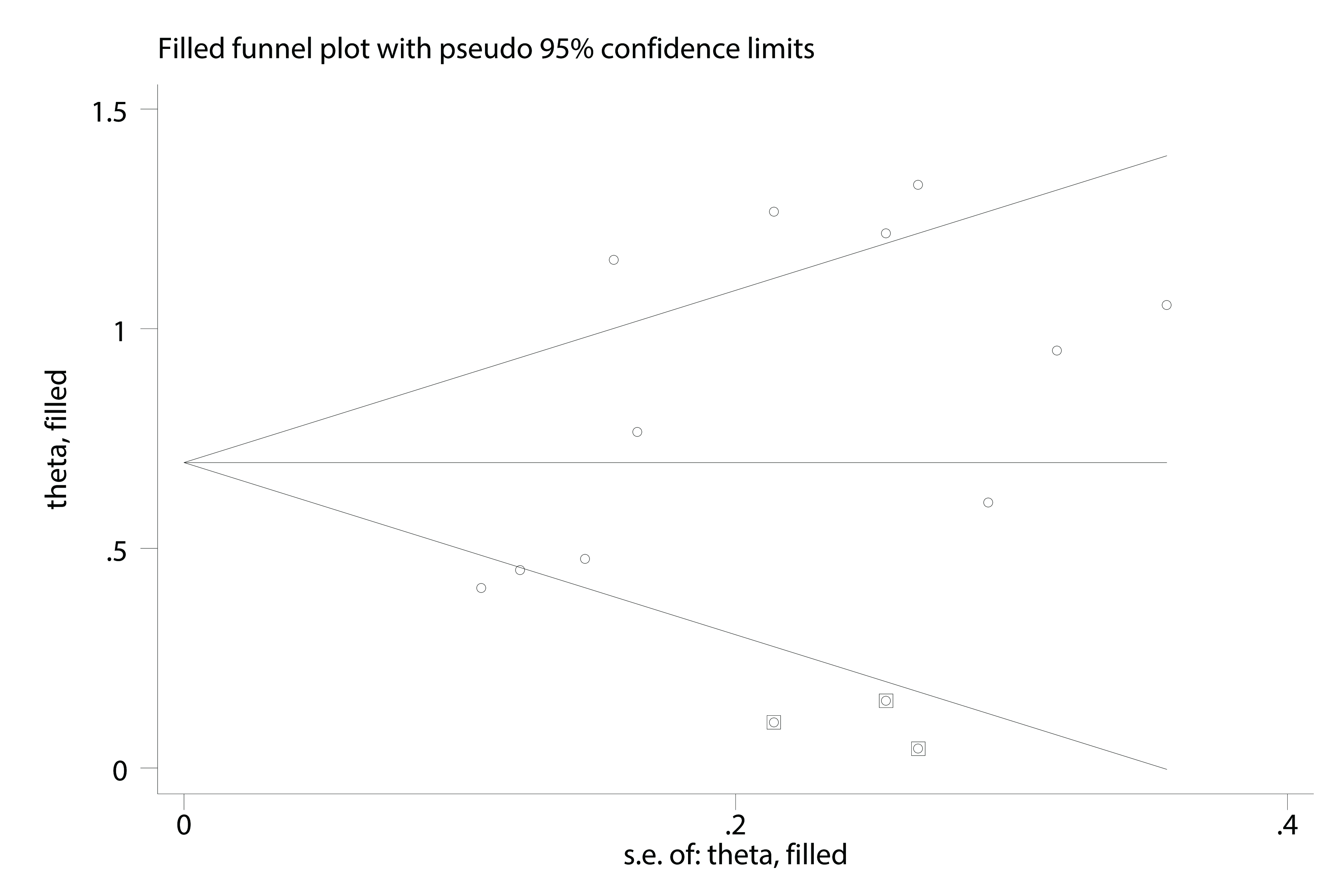

Supplement: Supplementary file 3 — Supplementary Material 3: The trim-and-fill method for the meta-analysis of stress hyperglycemia ratio and in-hospital mortality in acute myocardial infarction. [file 12872_2026_5845_MOESM3_ESM.tif]
